# Complete Bilateral Gemination of Maxillary Incisors with Separate Root Canals

**DOI:** 10.1155/2014/425343

**Published:** 2014-08-31

**Authors:** Lodd Mahendra, Sujatha Govindarajan, Muruganandhan Jayanandan, Shaik Mohammed Shamsudeen, Nalin Kumar, Ramasamy Madasamy

**Affiliations:** ^1^Department of Orthodontics and Dentofacial Orthopedics, Sri Venkateswara Dental College and Hospital, Thalambur, Near Navalur, Off OMR, Chennai, Tamil Nadu 603103, India; ^2^Department of Oral and Maxillofacial Pathology, Sri Venkateswara Dental College and Hospital, Thalambur, Near Navalur, Off OMR, Chennai, Tamil Nadu 603103, India

## Abstract

Developmental anomalies in the hard tissue are seen frequently in dental practice. Gemination and fusion are the most commonly encountered anomalies, and distinction between the two is always challenging. Gemination, also called double tooth, is an anomaly exhibiting two joined crowns and usually a single root. It represents an incomplete attempt of a single tooth germ to split. It is considered multifactorial in etiology, with genetic and environmental causes. This paper discusses a rare example of bilateral gemination (prevalence 0.04%) of maxillary central incisors with completely separated roots. Multidisciplinary care ensured a successful esthetic and functional outcome.

## 1. Introduction

Gemination is defined as an attempt of a single tooth bud to divide, with the resultant formation of a tooth with a bifid crown and usually a common root and root canal [1]. Gemination and fusion appear similar and are usually assessed by the number of teeth in the dentition. Gemination is a single enlarged tooth or joined tooth in which the tooth count is normal when the anomalous tooth is counted as one. Fusion is the combining of two tooth germs to form an enlarged tooth. Here the tooth count is one less, when the anomalous tooth is counted as one. Though it occurs in both dentitions, it has a higher prevalence in deciduous teeth, with a higher frequency in anterior maxillary region [2]. It is also found with a higher incidence in the lower jaw, with equal sex predilection [3, 4]. This anomaly leads to higher caries potential, malocclusion, changes in the dental arch shape, periodontal disease, hyper/hypodontia, and eruptive disturbance of successional tooth and creates poor esthetics [5].

## 2. Case Report

**Figure 1:**
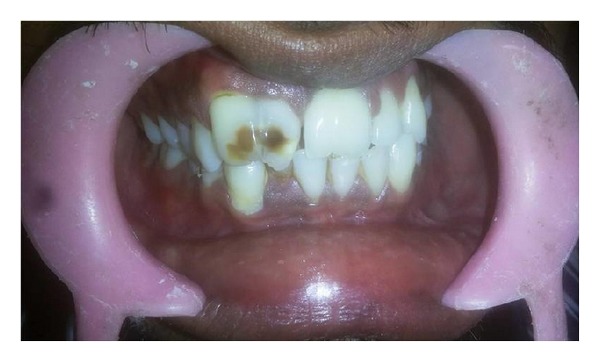
Clinical photograph—before treatment.

A 27-year-old male patient presented to the dental clinic with a complaint of enlarged maxillary incisors compromising esthetics (Figure 1). The patient was the second of 3 siblings of parents with no history of consanguinity. The patient appeared normal and healthy with no reported history of orofacial trauma.

**Figure 2:**
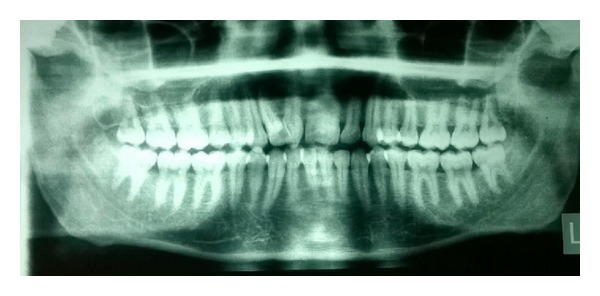
Orthopantomograph showing geminated maxillary central incisors with split roots.

**Figure 3:**
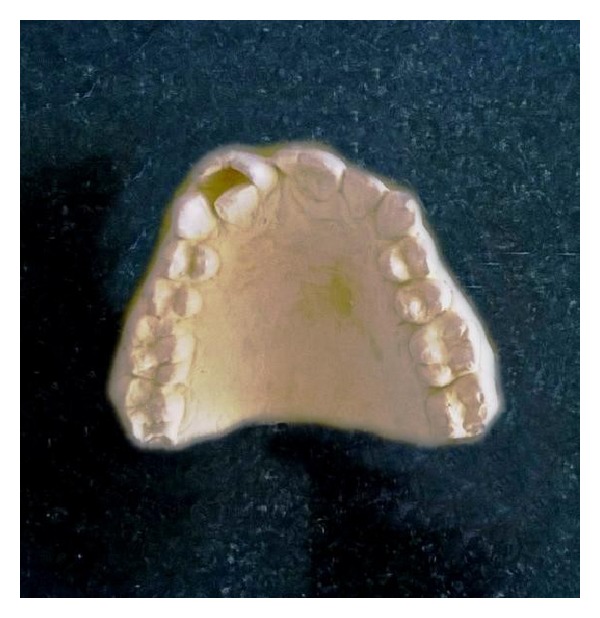
Study model.

On examination, the maxillary right and left central incisors appeared to have increased mesiodistal dimension with slight notching present in the incisal region extending through the labial cervical third. The right maxillary lateral incisors were palatally displaced while left lateral incisors were within the arch form. Dental caries was present in the mesial surface of the right maxillary central and lateral incisor. Orthopantomogram (OPG) and intraoral periapical (IOPA) radiographs revealed two completely separated roots of the right maxillary incisors with central notching presenting as a radiolucent line in the fused right maxillary incisor crown (Figure 2). The left incisor radiographically revealed a large crown and single root with notching represented as a radiolucent groove in the crown region. Two separate root canals were located in the widened root. Casts were made and options of treatment were discussed (Figure 3).

**Figure 4:**
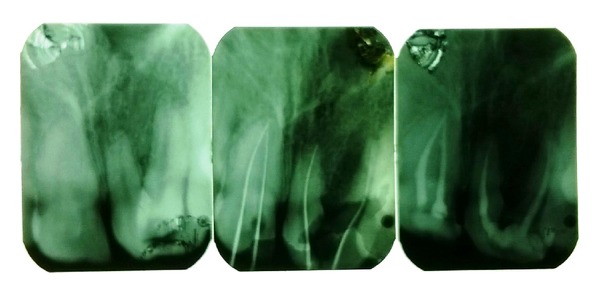
Intraoral periapical radiographs showing pre- and postendodontic treatment.

**Figure 5:**
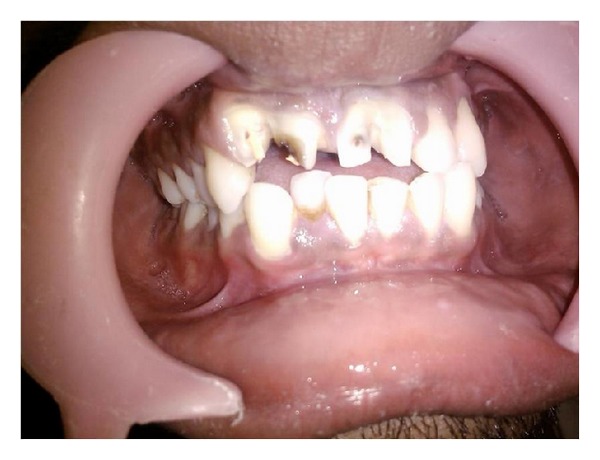
Clinical photograph of split crowns.

**Figure 6:**
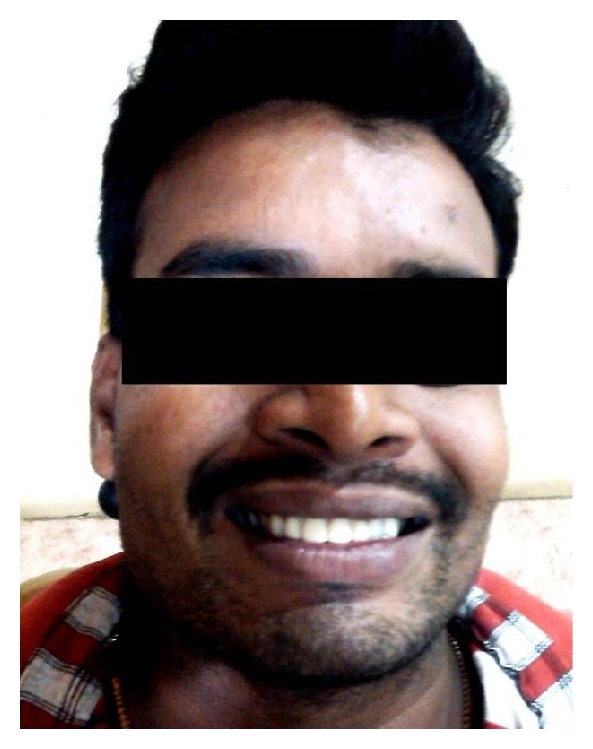
Clinical photograph—after treatment.

Since esthetics was the main concern of this patient, root canal treatment was done for the maxillary incisors (Figure 4). Since the right incisor had two separate roots, extraction of the palatally placed lateral incisor and splitting of the central incisors were done to create a lateral incisor (Figure 5). After uneventful root canal and extraction, postendodontic prosthetic rehabilitation was done. Placement of ceramic crowns established an acceptable smile (Figure 6).

## 3. Discussion 

Abnormalities in the form and number of teeth are congenital and appear in primary and permanent dentition. A disorder of growth or development in the anatomical structures that results in anything different from normal is called developmental anomaly [6]. Fusion and gemination are two different dental anomalies characterized by formation of clinically wide tooth [7]. Gemination occurs when a single tooth bud attempts to divide resulting in two completely or incompletely separated crowns with a single root or root canal [8]. Fusion occurs when there is union of two completely separate tooth buds. The terms “double tooth,” “double formations,” “joined teeth,” or “fused teeth” are often used to describe gemination and fusion [9].

When a single tooth is partially cleaved it is “true gemination” and when it is completely cleaved it is named “twinning.” Similarly when two tooth germs fuse during formation and show union of enamel and dentine they are termed “true fusion.” When union occurs in dentin and/or cementum, it is termed “late fusion” and a late fusion by cementum is termed “concrescence” [10]. Literature suggests that geminated teeth have single root canal and fused tooth has separate root canals. But an anatomically enlarged tooth with separated roots and bifid crown or a joined crown with an enlarged root and root canal is also a result of fusion and germination [11]. Our case presented with an enlarged bifid crown with separated roots on one side and fused roots on the other, a rare phenomenon. Another possibility is the fusion of two central incisors with two supernumerary teeth but that also is a rarity.

Unilateral gemination has a prevalence rate of 0.5% and 0.1% in deciduous and permanent dentition, respectively. Bilateral cases are seen in 0.01% to 0.04% in primary dentition and in 0.02% to 0.05% in permanent dentition [12]. Commonly affecting the anterior teeth with gemination more commonly in maxillary arch and fusion in the mandibular arch, this anomaly manifests itself from a minor notch in the incisal edge of an abnormally wide tooth to the appearance of almost two separate crowns in both the sexes with an equal sex predilection [13, 14]. Our case reported a bilateral gemination in the maxillary arch which is relatively rare.

In spite of various studies the etiology of gemination remains unknown. Evolution, trauma, heredity, and environmental factors are thought to play a role in germination [15]. Studies by Spouge have suggested that it may also result from traumatic disturbances to the developing tooth bud [16]. Hereditary tendency with mode of inheritance being either autosomal recessive or dominant affecting the dental lamina is also found to result in hyperdontia [17, 18]. Other reasons which are considered as the cause of gemination are nutritional deficiency, endocrine disturbances, infectious inflammatory processes, excessive ingestion of medicines, and ionizing radiation [12]. This anomaly may also be associated with syndromes such as chondroectodermal dysplasia and achondrodysplasia [19]. It is understood that gemination is caused by an interaction between a variety of genetic and environmental factors [20].

Clinically these patients present with a poor esthetics, a higher degree on caries, and periodontal problems. Due to the deep grooves present in the tooth these patients have a greater incidence of caries presentation. These grooves also lead to periodontal problems like pocket formation and gingival inflammation. When present in the primary dentition, exfoliation may occur depending on root resorption extents [21]. Other problems include occlusal disturbances and insufficient space in dental arch, leading to malocclusion. Esthetic rehabilitation is the most important requirement in these patients. But the caries and periodontal and occlusal disturbances are also taken into consideration for such patients and a multidisciplinary approach is required for establishment of proper esthetical and functional success of treatment. The aim of orthodontic treatment would be to retain the available teeth with proper alignment and occlusion. Periodontally, the presence of deep fissures and grooves extending subgingivally may lead to accumulation of plaque. When there is overlapping of the unusually large incisors accessibility becomes difficult and leads to further deposits and gingival inflammation. Presence of deep grooves also leads to high caries incidence and may require endodontic intervention [12]. Unimpressive esthetics is the major concern due to the unusually large tooth. Extraction followed by prosthetic rehabilitation may be considered as alternate line of treatment [22].

The treatment depends upon the patient requirement, the teeth involved, and the degree of involvement. If a primary tooth is involved the treatment depends upon the presence of the succedaneous tooth [23]. In our case esthetics was the primary concern of our patient. Two treatment options were considered. Firstly, following endodontic treatment of the geminated teeth, slicing and orthodontic management of the palatally placed lateral incisor to align the anteriors can be attempted.

Secondly, extraction of the palatally placed incisors and endodontic management of the geminated teeth may be done. This would be followed by slicing of central incisor to create a lateral incisor and capping of the endodontically managed teeth. Considering the duration of treatment the patient opted for the second option and the outcome was successful esthetically.

The presence of separated roots in the right central incisor created a lateral incisor after slicing. Oral hygiene instructions and regular recall appointments were advised for the patient. Multidisciplinary approach ensured functional and esthetical satisfaction.

## 4. Conclusion

The presence of gemination in primary or permanent dentition needs to be diagnosed and treated to ensure proper functional and esthetic satisfaction. The recognition and treatment of such anomalies are a challenge to the dentist. Gemination and fusion usually present as fused roots and rarely as separate roots as in our case. Diagnosis is based on the number of teeth present in the dental arch and the presence of separate roots favors endodontic restoration and prosthetic rehabilitation. Comprehensive approach is important to avoid further complications.
